# Supplementary material for: Exploration of Changes in Low-Income Latino Families’ Beliefs about Obesity, Nutrition, and Physical Activity: A Qualitative Post-Intervention Study
Source: Behav Sci (Basel). 2022 Mar 9;12(3):73. doi: 10.3390/bs12030073 (PMC8945531; doi:10.3390/bs12030073)
Supplement: Supplementary file 1 [file behavsci-12-00073-s001.zip › behavsci-1537784-supplementary.pdf]

Revised January 23, 2013

File S1：The Focus Group Moderator Guide

Research Objectives:

Objective 1: To assess for barriers to attempting or completing lifestyle changes recommended as a part of a treatment program that may be related to culture, income, or competing time demands in caregivers of Latino children from low-income families.

Objective 2: Among low-income, Latino families who participated in a community based, family oriented obesity treatment program how are parents' beliefs about obesity, nutrition, and physical activity related to their response to or evaluation of the program?

Objective 3: Among low-income, Latino families who participated in a community-based, family-oriented obesity treatment program, how is the level of acculturation* related to parents' beliefs about obesity, nutrition, and physical activity?

*Language being a proxy as a measure for acculturation those parents who only speak Spanish versus bilingual or English speaking only.

Objective 4: Among low-income, Latino families with an obese/overweight child who participated in a community-based, family oriented obesity treatment program, what is the relationship between the age of the index child and parents' beliefs about obesity, nutrition, and physical activity?

I. Welcome

A. Introductions and Consent

Good evening, thanks for joining us today. I'm Shauna and I am the Project Coordinator for the Healthy Living Program (HLP). Today we will be talking about the Healthy Living Program classes to get your feedback about them. This discussion will last about one and a half hours. We will be done by [TIME].

[INTERVIEWER GIVES PARTICIPANT CONSENT FORM]

Here is a consent form. Take a moment to review it and then we will talk about it.

[INTERVIEWER GIVES PARTICIPANTS 5 MINUTES TO REVIEW]

The consent form is an agreement to be a part of a discussion about the Healthy Living Program curriculum. You are joining this group because you want to, and you have the right to leave the group at any time.

Do you have any questions?

[MODERATOR PAUSES TO ANSWER ANY QUESTIONS]

If you agree to be in this discussion group, please sign the consent form.

[COLLECT SIGNED CONSENT FORMS AND HAND OUT BLANK ONES]

II. Ground Rules

Before we begin, let me mention a few things about how we usually conduct these groups:

1. My role is to guide our discussion by asking questions and to encourage everyone to share their thoughts and ideas. I won't be doing much talking, but may ask you to explain more or to give an example. Also, it's my job to see that everyone has a chance to voice their opinions, as well as to keep us moving along so that we have time to discuss all of the questions. So, at times, it might seem as though I am cutting you off. If so, please know that this is not meant to be rude, but rather to make sure that we have time to have a complete discussion of each question.

2. It's really important that everyone hear this: THERE ARE NO RIGHT OR WRONG ANSWERS!!! Each person's experiences and opinions are valid, and we want to hear a wide range of opinions on the questions we'll be asking. So, please speak up, whether you agree or disagree with what's being said, and let us know what you think.

3. Sometimes participants bring up sensitive issues during these discussions, and we want to be sure that everyone agrees that anything of a personal nature that is mentioned in this room will NOT be repeated to others outside of this discussion group. Can I see a nod from everyone showing me that you agree with this confidentiality ground rule? (If anyone is not willing to give their consent to confidentiality, they may be excused from

the group.)

4. Let me tell you about our recording process. As you can see, we are using two digital recorders to record our discussion. We usually record these focus groups because we want to capture everything that all of you say, and we simply can't write fast enough to get it all down. We'll be using only first names in the group discussion, and when we put together the results from all the groups, we won't include any names at all.
5. However, because we're recording, it is very important that we speak <u>one at a time</u>, so that the recorder picks up everything that is said. So, now that you know what our process is, is everyone OK with being recorded?

6. I'm also going to pass out note cards to each of you. I will collect these at the end of the session. If there is any feedback that you would prefer not to share out loud, that you prefer not to be recorded, or that you didn't have a chance to say, please write it down on these cards and then give them to us after the focus group is over. ***(Pass out note cards.)***

7. As a final reminder, please set any phone or other ringing devices to vibrate. If you need to take a call, please step out to do so and join us once you are done. The bathrooms are located (down the hall to the right). When we are done with our discussion each family will receive a gift card and dinner will be provided.

<u>Warm-Up:</u>

To get us started, I would like to go around the room and have each of you tell us

i. Your first name
ii. How many children you have?
iii. Their ages?
iv. What words do you prefer to use to describe/ identify your race or ethnicity?
v. A healthy change you feel proud of?

[MODERATOR PAUSES TO ALLOW TIME FOR INTRODUCTIONS]

Great, thanks for sharing.

Healthy Living Program Goals (HLP):

Throughout the rest of our discussion when I say "culture" I mean your heritage, which is a part of where you come from. A person's culture includes groups they feel a part of and the customs and traditions they follow.

1. What healthy behaviors have you/your family began or changed after being in the HLP?
2. What helped you make changes? **Probe:** Tell me how it made you feel to try to make changes?
3. Did anything prevent or stop you from making changes recommended?
4. What things did you/your family like about HLP?
5. What things did you/your family did not like about HLP?
6. How did the lifestyle changes (changes to what or how your family eats and the physical activity you do) that the Healthy Living Program recommended fit with your day-to-day life? **Probe**: In thinking about your culture how did the lifestyle changes that HLP recommended fit with your day-to-day life?
7. For some families money can be a barrier. How could we change the Healthy Living Program classes to make the health changes more accessible for your family?

Now we are going to move to a discussion with more specific questions on your thoughts about nutrition.

Nutrition Specific:

1. What are some of the good things that come from eating more fruits and vegetables per day?
2. What are some of the barriers or things that might stop you/your family from eating more fruits and vegetables per day?
3. Whose opinion do you trust when it comes to deciding what your family should eat? **Probe**: Why?
4. In thinking about the Healthy Living Program, how did the meals suggested fit with your culture regarding how families should cook and eat together?

5. What would other members of your family, for example your parents or grandparents, aunts, or uncles think about the types of changes that were recommended in regards to how you cook meals and things to eat? **Probe:** Why?

6. Who controls what food your child eats during the day? Who controls what food is in your house? How much control do you think you have over what your child/children eat?

7. In part of the classes, you met with Maria and talked about parenting techniques including discipline and setting limits. How do you think these classes helped you manage what your child/children eat?

[Provide list of Nutrition topics on poster. Give each participant a piece of paper with Nutrition topics listed on it with happy or sad faces.]

8. I have given each of you a list of the Nutrition topics we discussed in the Healthy Living Program classes. I'll read through them and as I do, please circle the happy face if you liked the topic or the sad face if you did not like the topic. (INCLUDE neutral response/neutral face?)

9. Which topics stand out? (Have a lot of happy faces or sad faces) [Discuss those topics mentioned by participants that stand out] Probe: How did ____(topic) make you feel?

We are now going to move on to discussion questions regarding physical activity.

<u>Physical Activity Specific:</u>

1. Before starting the Healthy Living Program what did you think of when a provider said that your child should be more active? **Probe:** Why?

2. What are some of the good things associated with making sure your child/your whole family is more active?

3. What types of goals did you set for your family around being more active? **Probe:** Why?

4. What types of things did your family come across that may have prevented you from completing the recommended goals by the HLP? **Probe:** How have you and your family tried to overcome those things?(May need to remind them of some of the recommended goals)

5. How much control do you feel you have over how much physical activity your child/children or the entire family is able to do?

6. How do things that you normally do in your culture, or family traditions help or get in the way of your family being more active?

7. What do others in your extended family, or close friends think about increasing physical activity?

[Repeat process with Physical Activity topics happy and sad faces for different topics]

Thank you for all your thoughts. Now we are going to move to our last topic of discussion, which is a discussion about ideas around an unhealthy weight.

Obesity Beliefs:

1. First, what does the term "unhealthy weight" mean to you? **Probe:** Why do you think that? Is there a term that you like better? Why?
2. Many of you were referred to the Healthy Living Program by your provider because he/she said that your child was an unhealthy weight. When you were told that, what were your thoughts? Probe: How did it make you feel? Did you believe him or her? Why or why not?
3. When you think of a healthy weight child who is preschool age how would you describe them? **Probe:** How about an older child who is in grade school (1st-6th grades) how would you describe them? What do they look like? (both physical and emotional attributes)
4. How would other people in your family such as the child's grandmother or aunt, or describe a healthy weight child? What about other parents you know in your community how would they describe a healthy weight child? **Probe:** Why do you think that is?
5. Whose opinion do you trust when it comes to whether your child is a healthy weight? **Probe:** Why?
6. How much control do you as the parent/guardian have over your child's weight? **Probe:** Why?
7. Some families have children that are still in preschool, others have only older children and some have both. How do you think the age of a child can make it easier or more difficult to control what he/she does whether it is what they eat or how active they are? **Probe:** In thinking about your child how did his/her age make is easier or more difficult to control eating and level of activity?
8. How do you think the age of your overweight child affected your decision to begin the HLP ?

That concludes all the questions I have, but before we finish I'd like to know from you if there is anything I've missed? Is there anything else that we should talk about that you think is important? Anything else you would like to share about the topics we discussed?

Thank you so much for being here tonight and for sharing your ideas with us! If there's anything further that you'd like to add, please feel free to write it on the note cards that we handed out and then give them to us before you leave.

Give money (and collect signatures). Collect notecards.
